# Novel Inorganic Nanomaterial-Based Therapy for Bone Tissue Regeneration

**DOI:** 10.3390/nano11030789

**Published:** 2021-03-19

**Authors:** Yu Fu, Shengjie Cui, Dan Luo, Yan Liu

**Affiliations:** 1 Fourth Clinical Division, Peking University School and Hospital of Stomatology; National Engineering Laboratory for Digital and Material Technology of Stomatology, Beijing Key Laboratory of Digital Stomatology, Beijing 100081, China; fuyu19880715@126.com; 2 Laboratory of Biomimetic Nanomaterials, Department of Orthodontics, Peking University School and Hospital of Stomatology, National Engineering Laboratory for Digital and Material Technology of Stomatology; Beijing Key Laboratory of Digital Stomatology, Beijing 100081, China; cuishengjie@pku.edu.cn; 3 CAS Center for Excellence in Nanoscience, Beijing Key Laboratory of Micro-nano Energy and Sensor, Beijing Institute of Nanoenergy and Nanosystems, Chinese Academy of Sciences, Beijing 100083, China

**Keywords:** inorganic nanomaterials, bone regeneration, nano hydroxyapatites, nano silica, metallic nanomaterials

## Abstract

Extensive bone defect repair remains a clinical challenge, since ideal implantable scaffolds require the integration of excellent biocompatibility, sufficient mechanical strength and high biological activity to support bone regeneration. The inorganic nanomaterial-based therapy is of great significance due to their excellent mechanical properties, adjustable biological interface and diversified functions. Calcium–phosphorus compounds, silica and metal-based materials are the most common categories of inorganic nanomaterials for bone defect repairing. Nano hydroxyapatites, similar to natural bone apatite minerals in terms of physiochemical and biological activities, are the most widely studied in the field of biomineralization. Nano silica could realize the bone-like hierarchical structure through biosilica mineralization process, and biomimetic silicifications could stimulate osteoblast activity for bone formation and also inhibit osteoclast differentiation. Novel metallic nanomaterials, including Ti, Mg, Zn and alloys, possess remarkable strength and stress absorption capacity, which could overcome the drawbacks of low mechanical properties of polymer-based materials and the brittleness of bioceramics. Moreover, the biodegradability, antibacterial activity and stem cell inducibility of metal nanomaterials can promote bone regeneration. In this review, the advantages of the novel inorganic nanomaterial-based therapy are summarized, laying the foundation for the development of novel bone regeneration strategies in future.

## 1. Introduction

**Figure 1:**
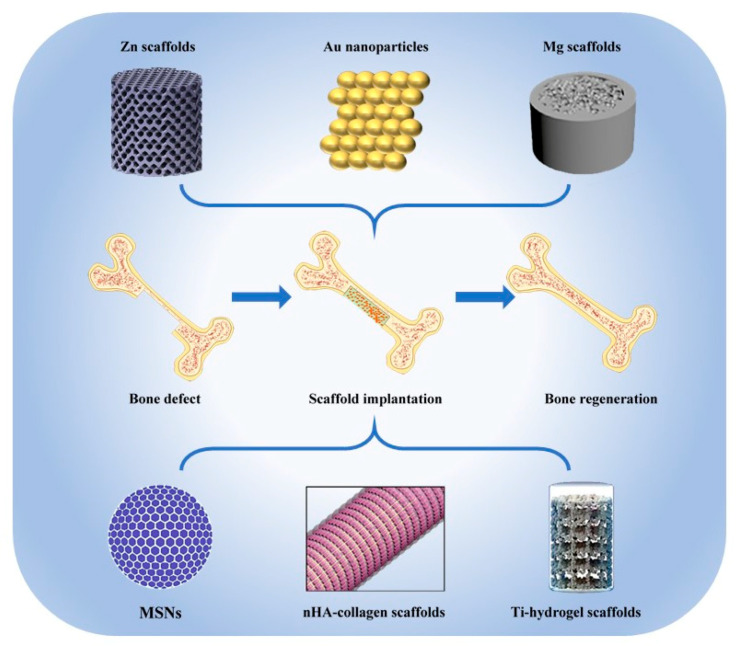
The main inorganic nanomaterials used for bone tissue regeneration [10,11,12,13,14].

Bone consists of inorganic minerals and organic matrix, and its highly hierarchical structure ensures excellent mechanical properties to withstand stress [1]. Although bone can heal itself through dynamic remodeling when suffering minor damage, bone lesions of critical size that cannot be cured spontaneously are quite common in daily life [2]. Until now, autologous transplantation is still the gold standard for the treatment of large bone defects [3]. However, the high incidence of donor sites mobility and the limited volume of autologous bone grafts limit the large-scale promotion of bone replacement surgery [4]. The ideal bone graft substitutes should not only imitate the extracellular matrix (ECM) of natural bone to achieve excellent biocompatibility, more importantly, they must provide strong mechanical support for the defect tissue. Inorganic nanomaterials possess better mechanical strength than natural and synthetic polymer scaffolds and can maintain stability for several weeks in vivo to support the bone healing process in the early stage of regeneration, making them the most promising candidate for bone graft substitutes. Calcium–phosphorus compounds, silica and metal-based materials are the most common categories of inorganic nanomaterials for bone defect repairing. Among them, nano hydroxyapatites (nHAs) are widely studied due to their high similarity with natural bone apatite [5]. Nano silica has been proven to establish hierarchical structure and promote bone regeneration through a biosilicification process [6]. For metal materials, traditional bulk metal scaffolds have been used for permanent and temporary orthopedic applications [7]. Recently, biodegradable metallic nanomaterials are able to be manufactured through a 3D printing technology, namely additive manufacturing (AM), to produce personalized orthopedic implants [8]. According to computer-aided design data, the mechanical properties, pore size, porosity and surface characteristics of the implant can be perfectly controlled [9]. In this review, the advantages and applications of the above three kinds of inorganic nanomaterials in bone tissue engineering will be reviewed (Figure 1).

## 2. Nano Hydroxyapatites

Natural mature bone is composed of minerals, type I collagen, water, small amounts of other collagen types, noncollagenous proteins and proteoglycans. The minerals mainly refer to thin plate-shaped carbonated calcium-deficient hydroxyapatites (50 nm × 25 nm in size, 1.5–4 nm thick), distributed along the collagen fibrils [1]. Hydroxyapatite (HA) has a Ca/P ratio of 1.67, higher than that of calcium-deficient hydroxyapatites, and is insoluble in vivo. HA is hard to degrade and has favorable mechanical properties and chemical binding ability, thus it is mostly used as a nanofiller in polymers to improve the mechanical strength or coating onto metallic implants to impart bioactivity [15].

### 2.1. nHA/Polymer Composites

**Figure 2:**
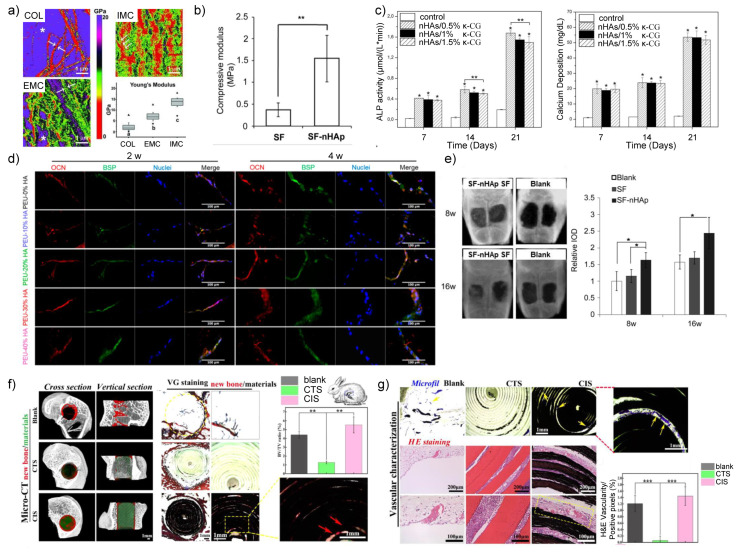
The mechanical and biological properties of nHA/polymer composites. (**a**) The highly ordered deposited nano hydroxyapatites (nHAs) provided intrafibrillarly mineralized collagen (IMC) with much higher Young’s modulus than that of pure collagen (COL) and extrafibrillarly mineralized collagen (EMC) [16]. (**b**) The addition of nHAs increased the compressive modulus of silk fibroin significantly [22]. (**c**) nHA/kappa-carrageenan (κ-CG) enhanced the alkaline phosphatase (ALP) activity and calcium deposition of human osteoblasts, regardless of the concentration of κ-CG [28]. (**d**) With the increase of nHAs content, the MC3T3-E1 preosteoblast cells expressed more osteocalcin (OCN) and bone sialoprotein (BSP) [24]. (**e**) The nHA/silk fibroin composites promoted the bone regeneration in rat calvarial defects [22]. The co-inspired scaffold (CIS) which consisted of nHAs and chitosan induced both new bone formation (**f**) and vascularization (**g**) compared with blank and pure chitosan [29]. *: *p* < 0.05, **: *p* < 0.01, ***: *p* < 0.001.

Inspired by the composition and structure of bone, researchers try to mineralize organic matrix with calcium phosphates, especially nHAs, which are the most stable and similar member with natural mineral phase in vivo. It has been shown that mineralization with nHAs enhanced Young’s modulus of type I collagen from 0.2–7.8 GPa to 11.1–16.3 GPa (Figure 2a) [16]. In addition to the improvement of mechanical properties, the mineralized fibrils showed rough surface topography and could promote the osteogenesis of mesenchymal stem cells (MSCs) in vitro and bone defect regeneration in vivo [17,18]. Even though chitosan had much higher compressive modulus (around 27 kPa) and compressive strength (around 36 kPa) compared to those of collagen (around 2 kPa and 3 kPa, respectively), nHAs were still able to significantly increase the mechanical properties of chitosan with compressive modulus of around 43 kPa and compressive strength of 40 kPa, respectively [19]. In another study, the Young’s modulus of pure chitosan was reported to be approximately 3.0 GPa, while that of nHA/chitosan composite increased to over 3.5 GPa [20]. Similarly, the addition of nHAs could elevate the ultimate compressive strength of chitosan both in wet and dry conditions and the fabrication methods of nHA/chitosan composites further affected the mechanical strength [21]. Silk fibroin is also a natural polymer with compressive modulus of 0.43 MPa, and after being assembled with nHAs, its compressive modulus became 4 times higher (Figure 2b) [22]. Aside from the abovementioned natural polymers, the synthetic polymers also need nHAs in the fabrication process. It has been shown that HA nanorods can increase the elastic modulus and strength of polycaprolactone by approximately 50% and 26%, respectively [23]. However, there was an exception when nHAs were used to fabricate nHA/poly (ester urea) composite scaffold through 3D printing technology. The addition of nHAs had no significant influence on compressive modulus, which may result from the balance between the reinforcement effect and nonoptimized nHA/poly(ester urea) interaction [24]. Unlike the controversial effect on polymers’ mechanical performance, nHAs can delay the degradation of poly(D, L-lactic acid)/poly(D, L-lactic- co-glycolic acid) blends [25]. Impressively, the effect of nHAs on the degradation rate of chitosan was even higher than that of nanobioglass, which may be attributed to the fine dispersion of nHAs in composites [26]. The addition of nHAs not only affects the mechanical performance and degradation rate of organic polymers, but also releases Ca^2+^ and PO_4_^3^^−^ ions and regulates osteogenesis and bone regeneration [27]. For instance, the appearance of nHAs in poly(ester urea) scaffold significantly increased the alkaline phosphatase (ALP) activity, bone sialoprotein (BSP) and osteocalcin (OCN) expression and calcium deposition of MC3T3-E1 preosteoblast cells (Figure 2d) [24]. Gonzalez Ocampo et al. also proved nHAs promoted the cell spreading, ALP activity and matrix mineralization of human osteoblasts seeded on kappa-carrageenan (Figure 2c) [28]. Furthermore, nHA/polymer composites can promote bone regeneration in both calvarial defect (Figure 2e) [22] and long bone defect models [29]. Based on the inspiration from cortical bone and nacre, Feng et al. fabricated a “brick and mortar” multilayer nHA-based scaffold, which induced not only osteogenesis, but excellent angiogenesis as well (Figure 2f,g) [29]. The effect of nHAs coating on metal-based nanomaterials will be briefly discussed in Section 4.

### 2.2. 3D Printed nHA-Based Inorganic Nanomaterials

**Figure 3:**
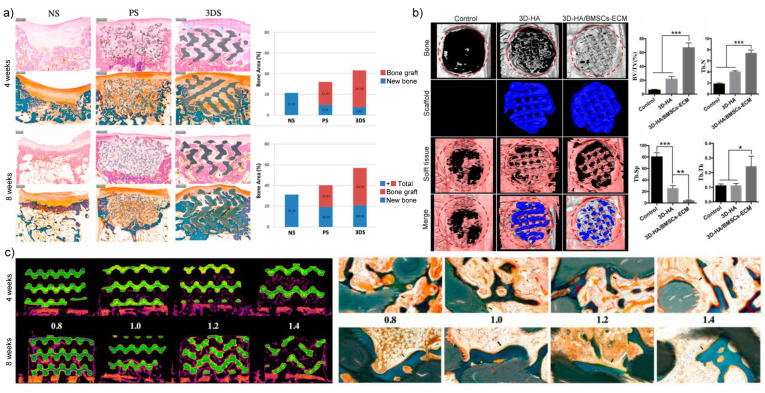
The properties affecting bone regeneration of nHA-based 3D printing scaffolds. (**a**) Histomorphometric examination demonstrated that the 3D printed HA/ tricalcium phosphate scaffold (3DS) had better bone reparation effect than both negative control (NS) and positive control (particle-type bone substitutes, PS) in beagle dog mandibular bone defects [30]. (**b**) Radiographic analysis showed that 3D hydroxyapatite/extracellular matrix (HA/ECM) composites promoted bone regeneration in rat calvarial defects after 12 weeks [34]. (**c**) Both radiographic and histological evaluations exhibited more new bone formation for 3D printed nHA-based scaffolds with higher pore size only at 4 weeks [35]. *: *p* < 0.05, **: *p* < 0.01, ***: *p* < 0.001.

The latest emerging 3D printing technology makes nHAs and other inorganic nanomaterials easy to shape and may help to solve the poor biodegradability and excessive mechanical properties of biomaterial scaffolds consisting only or mainly of nHAs. Compared with the commercially available particle-type bone substitutes OSTEON 3 (Genoss^®^), the 3D printed customizable HA/tricalcium phosphate scaffolds promoted more new bone formation (Figure 3a) [30]. Even using the same raw materials calcium phosphate cement and polyvinyl butyral, different solvent of polyvinyl butyral, such as ethanol and tetrahydrofuran could lead to quite different geometry, microstructure, mechanical properties and osteoconductivity [31]. nHA/polyvinyl butyral composite scaffold fabricated in ethanol had 2-fold higher ultimate tensile strength and 3.4-fold higher ultimate compressive strength and promoted the osteogenesis of human primary osteoblasts, compared with that made in tetrahydrofuran. In addition to internal structure, surface topography and chemical characteristics also contribute to the bone reparation effect. Wei et al. reported that the hexagon-like column array topography of 3D printed HA scaffold promoted the osteogenic differentiation of human adipose-derived stem cells [32]. Furthermore, this research group found that the surface modification with strontium ion substitution can enhance the effect of HA porous scaffold on osteogenesis [33]. Similarly, the ECM derived from bone marrow MSCs (BMSCs) modified the surface chemistry of 3D HA scaffold and then improved the ALP activity, osteogenesis-related mRNA expression and calcium deposition of BMSCs, which finally promoted the bone reparation in rat skull defects (Figure 3b) [34]. As for the pore size, the HA-based 3D printed scaffolds with 1.4 mm and 1.2 mm pore sizes can promote bone regeneration at 4 weeks in rabbit calvarial defects while decreasing the mechanical strength, compared with those scaffolds with 0.8 mm and 1.0 mm pore sizes. However, the effect of pore size on bone regeneration was diminished as time went on to 8 weeks (Figure 3c) [35]. Future research is required to explore better methods to accurately determine the topography of nHA-based 3D printing scaffolds.

## 3. Nano Silica

Nano silica is an important component of bioceramics, and it is widely applied in bone tissue engineering. According to the definition of the International Federation of Applied Chemistry, mesoporous materials have a pore size between 2 nm and 50 nm. Sol-gel method was initially used to synthesize regular pore structure in mesoporous silica nanoparticles (MSNs) with adjustable pore size [36]. The mesoporous structure offers good biocompatibility and biodegradability among inorganic nanomaterials [37]. Based on the exciting characteristics, MSNs are drawing more and more interest in the basic research of bone tissue engineering. MSNs serve as a kind of drug carrier with excellent release efficiency, and they also have the biological activity of promoting bone formation. Therefore, the researchers mainly focus on two aspects of modification: one is to modify the pore size adapted to functional factors that the mesoporous silicon material can load; the second is to explore different composite scaffold materials to improve the physiochemical property and bioactivity of MSNs.

**Figure 4:**
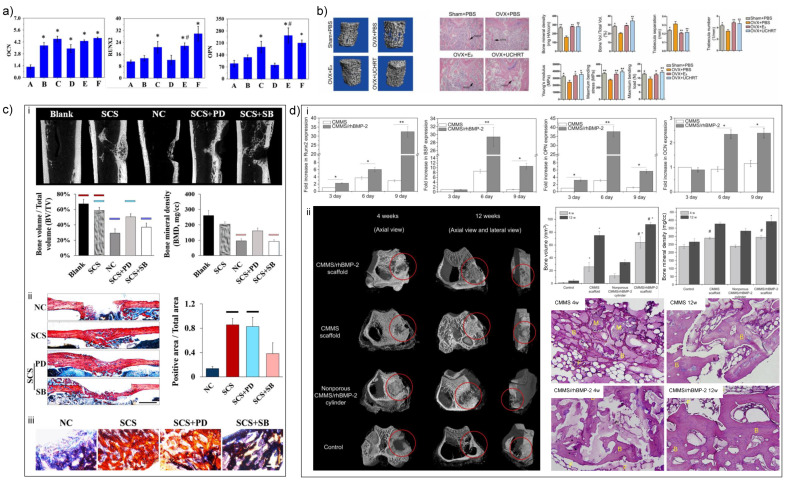
The application of nano silica in bone tissue engineering. (**a**) The effect of Si ions on osteogenesis-related gene expression of human bone marrow mesenchymal stem cells (BMSCs). A: blank control (DMEM); B: 10 μg/mL Si ions in DMEM; C: 50 μg/mL Si ions in DMEM; D–F: A–C solution plus 35 μg/mL dimethyloxaloylglycine, respectively. *: Comparison between blank and other groups; #: comparison between group B and E, C and F, respectively; *p* < 0.05 [13]. (**b**) Mesoporous silica nanoparticles (MSNs) assisted E2 sustained release, which prevented osteoporosis in vivo. OVX = ovariectomy; UCHRT = NaLuF_4_:Yb,Tm@NaLuF_4_@mSiO_2_-EDTA-E2 nanocomposites. *: *p* < 0.05, **: *p* < 0.01 [42]. (**c**) Nano silica incorporated collagen fibrils promoted bone regeneration in rat femoral defects. SCS = silicified collagen scaffold; NC: negative control; SCS + PD: silicified collagen scaffold plus PD098059; SCS + SB: silicified collagen scaffold plus SB203580; PD and SB are MAPK inhibitors. (**i**) Micro-CT scans and quantitative results. (**ii**) van Geison staining. Bar = 1 mm. (**iii**) Ponceau trichrome staining. Bar = 200 μm [52]. (**d**) The osteoconductivity of calcium/magnesium-doped rhBMP-2-incorporated MSN scaffold in vitro and in vivo. CMMS= calcium/magnesium-doped silica-based scaffolds. (**i**) rhBMP-2 increased the osteogenesis-related gene expression of rat BMSCs. (**ii**) CMMS/rhBMP-2 scaffold promoted bone regeneration in rabbit femoral defects as demonstrated by micro-CT analysis and histological evaluation. M: materials, B: bone, F: fibrous tissue. *: *p* < 0.05; #: *p* < 0.05 [53].

The mechanisms of MSNs that promote bone defect reparation are as follows: (1) the silicon ions released by hydrolysis can promote the expression of osteogenic-related genes in osteoblasts (Figure 4a) [13]; (2) the mesoporous structure helps the deposition of HA, which further promotes mineralization [38]; (3) MSNs induce efficient macrophage uptake and promote immunomodulatory effects, which are conducive to osteogenic differentiation [39]. Moreover, the efficient uptake is good for drug delivery in bone defect area in vivo. The efficiency of drug delivery relies on the pore size and superficial chemical modification. As the controllability of MSN fabrication based on various synthesis methods has already been testified to load different types of drugs, the characteristics of modified MSN-drug releasing system are used more and more often in basic research with or without combined scaffolds.

### 3.1. Drug Delivery Carrier

MSN loaded-drugs including traditional chemicals, protein and peptides and nucleotides exert positive effects on bone formation. Combining dexamethasone-loaded MSNs with mineralized porous biocomposite scaffolds induced bone regeneration by enhancing the osteogenic activity of host BMSCs [40]. Alvarez et al. demonstrated that ibandronate sodium-loaded MSNs incorporated into the collagen gel had continuous drug release around 10 days, which effectively inhibited the function of osteoclasts and promoted the osteogenic differentiation of MSCs [41]. 17-beta estradiol-loaded MSNs with EDTA modified surface significantly enhanced the efficiency of hormone therapy for osteoporosis (Figure 4b) [42]. Furthermore, injectable miR222/MSN/aspirin hydrogel promoted mandibular bone regeneration and induced neurogenesis in the defect area [43]. Therefore, the effectiveness of drug delivery of MSNs is demonstrated by numerous studies and is a promising strategy for drug-MSN-scaffold-induced bone regeneration. To acquire maximum loading capacity of drugs, researchers modified the structure of MSNs with rough or hydrophilic surface, which enhanced the adhesion of the target drug [44]. Based on previous studies, hollow structure has enough space for drug loading and storage, thus hollow mesoporous silica materials provide new insight for mesoporous silica-based drug delivery [45]. However, the simple modification of surface or pore size of MSNs is not sufficient to realize precise releasing system. Therefore, the incorporation of biomacromolecules (organic polymers, macrocyclic molecules, etc.) and MSNs enhances the efficacy of MSN-drug releasing system.

### 3.2. Modification of MSN-Based Scaffolds 

In order to acquire enough strength, proper morphology and enhanced bioactivity of MSNs for bone formation, the composites of MSNs and organic/inorganic scaffolds have been fabricated. Considering the importance of nHAs during biomineralization, numerous studies have adopted the combination of MSNs with nHAs and testified to the synergistic effects on bone formation. He et al. showed that MSN/nHA composites enhanced the adhesion and proliferation rate and osteogenesis-related gene expression of rabbit BMSCs and new bone formation in rabbit femur defects, compared to MSNs or nHAs alone [46]. Similar co-enhancement effect on osteogenic differentiation of MG-63 cells was found by Shuai et al. [47]. Furthermore, the existence of nHAs endowed discontinuous pore surface of MSNs, which induced rapid drug releasing of ciprofloxacin from 32% to 93% in 24 h [48]. Along with nHA-silica nanomaterials, the natural components of extracellular matrix can induce an increase of cell–scaffold interaction and result in better biocompatibility and bioactivity of nano silica. In turn, the application of silica reinforced the mechanical strength, enhanced the water uptake capacity, and fastened degradation rate of matrix scaffolds [49], which benefited repair of large-scale bone defects. Thus, the application of matrix components as silica-based scaffolds is attracting the interest of researchers. Gaihre et al. reported that injectable nano silica–chitosan microparticles performed significant enhancement of ALP activity during osteogenic induction of osteoblasts [50]. In addition, collagen-alginate-nano-silica microspheres improved the osteogenic potential of human osteoblast-like MG-63 cells by promoting the expression of OCN and BMP-2 [51]. Intrafibrillar silicified collagen scaffold promoted in situ bone regeneration via p38 signaling pathway in monocytes and recruited host MSCs (Figure 4c) [52]. Therefore, it is a promising method to fabricate nano silica with natural polymer scaffolds for bone regeneration applications. 

Furthermore, some studies focus on doped metal ions in nano-silica-based materials. Dai et al. synthesized magnesium-doped mesoporous silica materials containing recombinant human bone morphogenetic protein-2 (rhBMP-2) with macroporous and mesoporous structure using polyurethane foam as a template (Figure 4d) [53]. This magnesium-doped silica material induced osteogenic differentiation of rat BMSCs in vitro and ectopic osteogenesis in vivo and also showed a good repair effect in a rabbit femoral defect model with diameter of 5 mm and depth of 5 mm. Shi et al. demonstrated that copper-doped MSNs induced robust immunomodulatory effects of murine-derived macrophage cell line RAW 264.7 and promoted osteogenesis of human BMSCs [39]. Recently, rare earth elements, such as lanthanum- [54], europium- [55] and gadolinium-doped [56] MSNs showed the exciting ability in osteogenesis of BMSCs and bone regeneration in rat skull bone defects. Despite the positive results in osteogenesis in vitro, the application of metal ions in nano silica materials still need more in vivo evidence to demonstrate the effectiveness and biosafety of such modification of nano silica.

## 4. Metallic Nanomaterials

Most traditional bulk metals exhibit much higher mechanical properties and worse bioactivity than natural bone, which may lead to bone resorption and poor osteointegration and osteogenesis [9]. Surface modification at nanoscale would help to improve the surface topography and chemistry of metal implants. In order to balance the gap in mechanical properties, the researchers tried to build a micro-nano structure to increase the porosity of metal materials to more than 50% [57]. In the meanwhile, porous materials can promote the penetration of cells and nutrients and the regeneration of bone and blood vessel when the pore size exceeds 300 μm [58]. In addition, the metallic nanomaterials are preferably biodegradable, which means they are expected to gradually corrode in vivo, and the corrosion products are metabolized or absorbed by cells and/or tissues. After the defects are repaired, the implants are completely dissolved without residue. In the following sections, four widely studied metal nanomaterials are introduced. 

### 4.1. Ti-Based Nanomaterials

#### 4.1.1. Nanoscale Surface Modification of Ti-Based Biomaterials

Titanium (Ti)-based biomaterials are widely used as permanent implants in orthopedic surgery and dental implantation because of their high load-bearing properties, nondegradability and good biocompatibility [59]. Due to the superior corrosion resistance of bulk Ti and its alloy, they often exhibit slow biological response, low osseointegration rate and lack of antibacterial properties. Nanoscale surface modification strategies, such as coating and doping, are effective means to solve the above problems and endow the implants with functionalization. In order to enhance the osteoconductivity, Ding et al. mixed strontium-incorporated lysozyme solution with Ti substrates and spontaneously formed a 2D nanofilm which could promote the ALP activity and osteogenesis-related genes expression of BMSCs [60]. The sustained release of Sr^2+^ from lysozyme nanofilm further facilitated osteointegration of Ti implants in rat femur bone defect model. Similarly, nano-graphene oxide (GO) was deposited on Ti surface through ultrasonic atomization spraying technique. This nano-GO coating induced the osteogenic differentiation of rat BMSCs via FAK/P38 signaling pathways and further accelerated bone regeneration in vivo [61]. Furthermore, lanthanide mineral-substituted hydroxyapatite nanorods were coated on Ti substrates by electrophoretic deposition method to mimic the topography and composition of natural bone [62]. The lanthanide mineral-substituted hydroxyapatite nanorods modified Ti implants had better osteogenic effect in vitro and bone regeneration effect in rat tibia defects. Compared to the above-mentioned inorganic coatings, human MSC-derived extracellular vesicles (MSC-EVs) were difficult to be anchored onto pristine Ti. Chen et al. successfully self-assembled human MSC-EVs onto biotin-doped polypyrrole titanium, which exhibited osteoinductivity in nude mice ectopic bone formation model with the help of osteoinductive miRNAs tested in MSC-EVs [63]. As for the antimicrobial activity, Zhang et al., inspired by the adhesion mechanism of mussel, developed a novel silicon-doped calcium phosphate composite coating (Van-pBNPs/pep@pSiCaP) loaded with vancomycin on porous Ti scaffold via modified surface mineralization process [64]. The functionalized biomimetic Ti scaffold can prevent the adhesion and proliferation of *staphylococcus epidermidis*. In addition to antibiotics, metal ions also have bactericidal activity. Huang et al. fabricated Cu-containing micro/nanotopographical bioceramic surface through micro-arc oxidation. The subsequent hydrothermal and final heat treatment can assist Ti implants to induce proinflammatory M1 macrophage polarization via Cu-transport signaling pathway and enhance bacterial phagocytosis. [65]. In addition to chemical antibacterial, the researchers prepared zinc oxide@collagen type I coating to achieve a broad-spectrum antibacterial effect through the photothermal effect of zinc oxide [66]. Surface modification materials can also cooperate with metal matrix to promote bone tissue repair. Fu et al. proved that the silicon-doped hydroxyapatite coating could demonstrate not only enhanced osteogenesis, but also promote angiogenesis [67].

#### 4.1.2. Additive Manufacturing of Ti-Based Biomaterials

**Figure 5:**
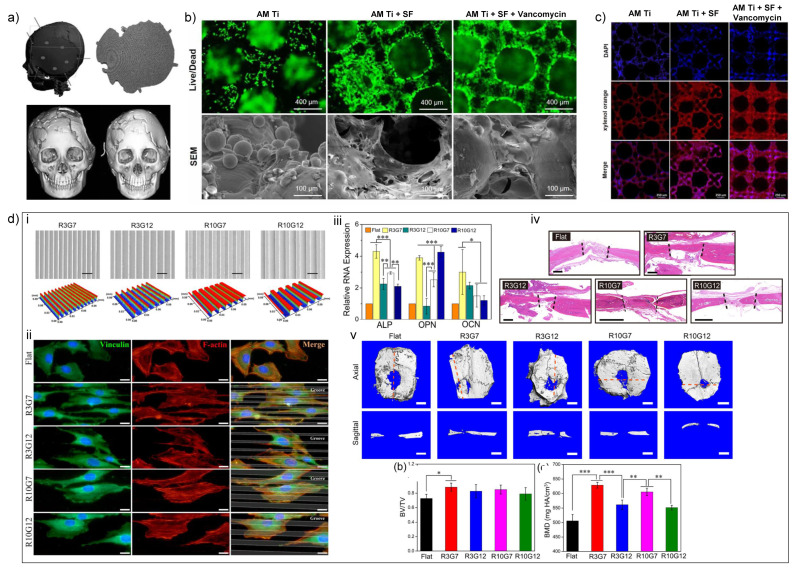
The biological properties of AM Ti-based implants. (**a**) Custom-made Ti-6Al-4V-ELI AM implants helped to reconstruct the skull defect of 21 patients [74]. (**b**) Silk fibroin and vancomycin coating promoted the survival of MC3T3-E1 cell line seeded on 3D printed Ti implants [82].(**c**) Silk fibroin and vancomycin coating promoted the calcium deposition of MC3T3-E1 cell line seeded on 3D printed Ti implants [82]. (**d**) The micropattern of Ti substrates induced osteogenesis. (**i**) SEM images and 3D surface profile of different micropatterns. (**ii**) MC3T3-E1 preosteoblasts aligned along the ridges in R3G7 micropattern. (**iii**) The R3G7 microgroove pattern promoted osteogenesis of MC3T3-E1 cells. (**iv**) and (**v**) The R3G7 microgroove pattern induced bone regeneration in rat skull defects [80]. *: *p* < 0.05; **: *p* < 0.01; ***: *p* < 0.001.

Traditional bulk Ti implants have the advantages of easy commercial availability and wide clinical acceptance, but their much higher stiffness than natural bone may cause stress shielding, leading to bone resorption and implant failure [68]. Although investigators are committed to improving the surface topography and chemical structure of bulk Ti implants, the porous structure, essential to the mechanical properties of natural bone, could not be simulated through traditional manufacture methods. The emergence of AM technologies is expected to solve this problem fundamentally. The computer-controlled and bottom-up fabrication process of AM technologies can more easily realize the control of chemical composition and micro-nano structure, so as to obtain better biomechanical properties to promote bone regeneration [69]. The elastic modulus of Ti–6Al–4V–10Mo [70], Ti–35Nb [71] and Ti–50Ta [72] (in wt%) nanomaterials prepared by 3D printing was significantly reduced to 73–84.7 GPa. The 3D printed, customized Ti implants have been used in craniofacial and orthopedic applications [73]. A clinical study involving 21 patients demonstrated good fixation between bone and custom-made 3D-printed Ti implants (with surface area ranging from 12,146 to 24,980 mm^2^) and satisfactory skull-shape symmetry without any complications during the 6 to 24 months follow-up period (Figure 5a) [74]. For tongue cancer excision with a tumor recurrence, Lee et al. utilized Ti-6AL-4V-ELI medical grade powder to fabricate a Ti implant with four dental implants based on computer-aided design data and restored the facial symmetry and occlusion at 5 months after surgery [75]. Impressively, Ti-6AL-4V-based AM implant with a total volume of 30.7 cm^3^ was able to successfully replace the complicated bone defects of distal tibia and foot caused by motor vehicle collision, and the patients could basically return to normal life and walk without ambulatory aids after 6 months [76]. Although there are still early and late complications, cranioplasty based on AM Ti implants has a higher success rate compared with other techniques [77]. In order to modify porous Ti nanomaterials based on AM, geometrical cues and surface structure design were investigated. As the porosity of AM Ti implants increased from 55.51% to 76.14%, the yield compressive strength decreased from 186.05 ± 1.85 MPa to 51.95 ± 0.62 MPa, and the elastic moduli decreased from 6.74 ± 0.47 GPa to 0.98 ± 0.03 GPa [78]. Nonetheless, the sharp decline of mechanical strength did not affect the bone regeneration, which may be attributed to the optimum porosity. Interestingly, the regeneration results of implanted non-load-bearing bones (such as the skull) were much worse than those of load-bearing bones such as the femur, which indicated the importance of stress stimulation [78]. In contrast, Maietta et al. demonstrated that with the help of finite element analysis, the Ti-6AL-4V architectural characteristics, such as pore size and shape, could be changed without significant alteration in mechanical properties [79]. For biological applications, Zhu et al. proved that shape- and size-controlled microgroove-patterned Ti surface structure manufactured by a combination of photolithography and inductively coupled plasma-based dry etching was beneficial to osteogenesis and bone regeneration [80]. Specifically, R3G7 (ridge width of 3 μm, groove width of 7 μm and depth of 2 μm) was the most effective micropattern to promote the osteogenic differentiation of MC3T3-E1 cells and bone regeneration in rat calvarial defects (Figure 5d). In addition to porosity and micropattern, roughness also plays an important role. Saruta et al. found that submicro-rough surface (with roughness of 24 ± 1.2 nm) promoted the attachment, proliferation and calcium deposition of osteoblasts, while micro-rough surface (with roughness of 123 ± 6.15 nm) had the strongest bone–implant integration in rat femurs [81]. Surface biofunctionalization is also well investigated to modify AM porous Ti-based nanomaterials. Several kinds of coating including silk fibroin, calcium phosphate and tricalcium phosphate can promote bone integration and regeneration both in normal [64,82] and osteoporotic bone defect models [83] (Figure 5b,c). The vancomycin coating helps to prevent the bacteria colonization caused by the high surface area to volume ratio of porous Ti implants [82]. On the other hand, a high surface area/volume ratio is beneficial for drug sustained release, such as BMP-2 and silver nanoparticles [84]. 

As mentioned above, AM Ti-based nanomaterials would have broad application aspects in bone tissue regeneration. The excellent chemical stability of titanium makes it difficult to degrade in vivo. Although titanium-based biomaterials are still the mainstream of current clinical applications, the developments of biodegradable metallic nanomaterials are promising and necessary. 

### 4.2. Mg-Based Biomaterials

#### 4.2.1. Nanoscale Surface Modification of Mg-Based Biomaterials

Magnesium is the most studied biodegradable metal ion. It has been confirmed that magnesium ions can induce bone marrow MSCs to differentiate into osteoblast lineage through canonical Wnt signaling pathway [85]. In distraction osteogenesis model, high-purity magnesium pins promoted angiogenesis and bone consolidation [86]. Moreover, the elastic modulus of magnesium is much lower than that of Ti, preventing bone resorption and implant failures induced by stress shielding. Additionally, the excellent degradability of Mg makes it an excellent temporary bone fixation device and possible low load-bearing bone substitute [87]. Degradability is a double-edged sword. In most cases, pure Mg implants degrade too fast to fully support new bone formation and the rapid release of hydrogen may interfere with the local microenvironment and hinder bone regeneration. Therefore, alloys of magnesium with calcium [7], zinc [7], strontium [88] and rare earth (RE) elements [89] have been developed to solve the above-mentioned problems. A long-term clinical study has confirmed the early bone healing and the complete replacement of Mg-5wt%Ca-1wt%Zn alloy by new bone in the late stage [7]. Fifty-three patients with hand and wrist fractures were involved in this study. All the patients returned to normal life with no sign of pain and the Mg-5wt%Ca-1wt%Zn implants were degraded, as confirmed by radiographic examination within 1 year, demonstrating the controlled degradation of this kind of Mg-based alloy. Furthermore, in order to reduce the corrosion rate of Mg, various nanoscale surface coating strategies have been applied to provide Mg-based alloys with a protective layer [90]. Zhang et al. developed calcium-phosphate-coated Mg−Zn−Gd scaffolds via chemical deposition method, which could not only repair rat cranial defects of critical size, but also promote osteogenesis, angiogenesis and the production of neuropeptide calcitonin gene-related peptide from trigeminal neurons in the orbital bone defect model of beagle dog [91]. Similarly, polycaprolactone and nHA nanocomposites dual coating via dip-coating and electrospinning could induce bone regeneration in rabbit femoral defects [92]. Kang et al. fabricated poly(ether imide)–SiO_2_/nHA-coated porous Mg scaffold through dip-coating technique, and repaired the bone defect in the femoropatellar groove model of rabbits due to the proper corrosion rate and enhanced mechanical strength of hybrid scaffold [12]. In addition, the surface modification by inorganic nanomaterials can also bring new functions to the implanted scaffold. For example, a functionalized 70-nm-thick TiO_2_/Mg_2_TiO_4_ nanolayer was fabricated by plasma ion immersion implantation technique on WE43 Mg-based alloy. The TiO_2_/Mg_2_TiO_4_ coating can promote osteogenesis of MC3T3-E1 cells and induce twice and six times higher levels of new bone volume (175%) than those of pristine WE43 alloy (88%) and blank control (28%) in rat femoral defect model. In the meanwhile, TiO_2_/Mg_2_TiO_4_ nanolayer suppressed bacterial infection and controlled the degradation behavior [93]. As for the immumodulatory effect, the combination of fibrinogen and magnesium can lead macrophages to M2 polarization and further promote osteogenic differentiation of MSCs [94]. In some special bone defect models (such as osteoporotic fracture), biodegradable Mg-based implants may serve as a sustained drug delivery system. The calcium phosphate nanocoating ensured the suitable degradation rate and structural integrity of Mg-based alloy, and the co-delivered magnesium degradation products and zoledronic acid modulated bone formation and resorption [95].

#### 4.2.2. Additive Manufacturing of Mg-Based Biomaterials

Compared to bulk Mg-based biomaterials, the AM of biodegradable Mg implants is still quite difficult due to the intrinsic properties of magnesium. Salehi et al. developed a novel 3D printing technique followed by sintering process to fabricate the Mg-5.9Zn-0.13Zr alloy with high precision at nano- and micron-scale. This AM Mg-based alloy possessed an average pore diameter of 15 μm, compressive strength of 174 MPa and elastic modulus of 18 GPa, which were quite similar to those of human cortical bone [96]. Until now, only a few research groups fabricated Mg alloys through selective laser melting technique [97] and investigated their mechanical and degradation properties. The biological performances of AM Mg-based alloys, for instance biocompatibility and regenerative effect in vivo, need to be further investigated.

### 4.3. Zn-Based Biomaterials

Zinc is an essential element for humans and plays an important role in many physiological activities. Zinc ions can enter human MSCs with the help of TRPM7 and GPR39, and then activate cAMP-PKA pathway, thereby ultimately enhancing cell survival/proliferation, differentiation, ECM mineralization and osteogenesis [98]. The degradation rate of Zn-based biomaterials is slower than that of Mg, and matches the tissue healing speed. The mechanical properties of Zn materials are between Ti and Mg, similar to natural bone [99]. Biodegradable Zn alloys, such as Zn-0.8%Li- (Mg, Ag), Zn–2Ag–1.8Au–0.2V and Zn-1Ag have better mechanical properties, corrosion rates and antibacterial activities than pure Zn implants, while only certain alloys have modest biocompatibility [100]. After the first published work in 2017 [101], a growing number of AM Zn-based nanomaterials appeared. The mechanical properties of AM porous Zn are similar to those of cancellous bone, and the degradation rates allow satisfactory bone substitution [102]. Although Zn exhibits mild cytotoxicity, it is still acceptable [103]. Like Mg, the biological evaluation of Zn-based biomaterials, especially AM porous Zn and alloys, remains blank.

### 4.4. Au-Based Biomaterials

Gold nanoparticles (AuNPs) have been widely investigated in biomedical applications, especially in antitumor therapy and bone regeneration therapy, due to their good biocompatibility, photothermal stability and near-infrared absorbance. The size and morphology of gold nanomaterials are important factors affecting their biological functions. Celentano et al. have successfully synthesized stable ultra-small gold nanoparticles, anisotropic gold nanoflowers and twisted gold nanorods by a simple green method and confirmed the biocompatibility of these AuNPs [104,105,106]. The addition of AuNPs into poly(methyl methacrylate)-based bone cement can significantly improve the punching performances while maintaining stable compressive properties [107]. In addition to better mechanical properties, as an efficient photothermal agent, AuNPs can eradicate residual tumor cells after the solid tumor is removed through photothermal therapy [108]. The theory of photothermal therapy can also be used to treat osteomyelitis. Gold-nanocage-containing aspirin can convert laser light into heat and realize controlled release of aspirin, which can perform anti-inflammatory effects on monocytes and promote bone regeneration after the monocytes eliminate infection [109]. Similarly, Sanchez-Casanova et al. entrapped heat-activated transgenic cell constructs in near-infrared-responsive hydrogels containing AuNPs, which can conditionally produce BMP-2 and promote bone regeneration in vivo [110]. Despite the above progress, it is still necessary to clarify how to co-assemble AuNPs and biomolecules and form biomimetic hierarchical scaffolds to achieve better outcomes in the field of bone regeneration therapy.

## 5. Concluding Remarks and Future Perspectives

Trauma, infection, tumor, degenerative and congenital diseases are the major causes of excessive bone defects. The current bone tissue engineering is trying to develop new materials to replace autologous bone grafts to achieve bone regeneration. Novel therapies based on inorganic nanomaterials, providing excellent mechanical properties and abundant physical/chemical/biological functions, are complementary to natural biomacromolecules and polymeric-based materials (Table 1). The development of inorganic nanoparticle/polymer composites can effectively integrate the respective advantages of inorganic and organic phases, bringing unlimited possibilities for the development of novel bone substitute materials. Biomimetic mineralization utilizing nano hydroxyapatites and nano silica is currently one of the most successful organic material modification processes. Furthermore, with the progress of technology, precisely designed porous inorganic nanomaterials appear to have lower stiffness and elastic modulus than traditional bulk materials, making them much closer to natural bone. Additive manufacturing is the most promising technology to realize computer-aided design and personalized customization. The 3D printed nHA-based nanomaterials and Ti-, Mg-, Zn-based metals would be able to act as temporary fixations or permanent implants according to their own characteristics, and eventually be replaced by regenerated bone tissue. Finally, inorganic nanoparticles are excellent carriers of drugs, growth factors and genes. nHAs, MSNs and AuNPs are commonly used to carry drugs into scaffolds and realize sustained release with the controllable degradation of scaffolds. 

## Figures and Tables

**Table 1:** The advantages and drawbacks of novel inorganic nanomaterial.

Type	Advantages	Drawbacks
nHA/polymer composites	(1) Enhanced mechanical performance of polymers [16,19,20,21,22,23] (2) Delayed degradation rate of polymers [25,26] (3) Enhanced osteogenesis [24,27,28,29]	Not custom-made
3D printed nHA-based inorganic nanomaterials	(1) Customized [30] (2) Flexible mechanical and biological properties [31,32]	Immature design and manufacturing methods [35]
MSNs	(1) Good biocompatibility [37] (2) Good biodegradability [37] (3) Sustained release of silicon ions [13], drugs [40,41,42], cytokines and miRNAs [43] (4) Immunomodulatory effects [39]	Seldom used alone in bone regeneration
Ti-based nanomaterials	(1) High load-bearing properties [59] (2) Good biocompatibility [59] (3) AM Ti nanomaterials could change the exorbitant elastic modulus of traditional Ti materials [69,70,71,72] and replace complicated bone defects [74,75,76].	(1) Nondegradability [59] (2) Poor biological response and anti-bacterial properties [59]
Mg-based biomaterials	(1) Biodegradability [86] (2) Osteoinductivity [85,86]	(1) Fast degradation and hydrogen release rate (2) Low elastic modulus for load-bearing bone defects [87] (3) Immature AM technology of Mg-based biomaterials
Zn-based biomaterials	(1) Biodegradability and suitable degradation rate (2) Suitable mechanical properties similar to natural bone [99]	(1) Mild cytotoxicity [103] (2) Immature AM technology of Zn-based biomaterials
AuNPs	(1) Good biocompatibility [108] (2) Photothermal stability [108] (3) Near-infrared absorbance [108]	(1) Seldom used alone in bone regeneration (2) The co-assembly of AuNPs and biomolecules lacks hierarchical structure
